# Sustained Effectiveness of Pfizer-BioNTech and Moderna Vaccines Against COVID-19 Associated Hospitalizations Among Adults — United States, March–July 2021

**DOI:** 10.15585/mmwr.mm7034e2

**Published:** 2021-08-27

**Authors:** Mark W. Tenforde, Wesley H. Self, Eric A. Naioti, Adit A. Ginde, David J. Douin, Samantha M. Olson, H. Keipp Talbot, Jonathan D. Casey, Nicholas M. Mohr, Anne Zepeski, Manjusha Gaglani, Tresa McNeal, Shekhar Ghamande, Nathan I. Shapiro, Kevin W. Gibbs, D. Clark Files, David N. Hager, Arber Shehu, Matthew E. Prekker, Heidi L. Erickson, Michelle N. Gong, Amira Mohamed, Daniel J. Henning, Jay S. Steingrub, Ithan D. Peltan, Samuel M. Brown, Emily T. Martin, Arnold S. Monto, Akram Khan, Catherine L. Hough, Laurence W. Busse, Caitlin C. ten Lohuis, Abhijit Duggal, Jennifer G. Wilson, Alexandra June Gordon, Nida Qadir, Steven Y. Chang, Christopher Mallow, Carolina Rivas, Hilary M. Babcock, Jennie H. Kwon, Matthew C. Exline, Natasha Halasa, James D. Chappell, Adam S. Lauring, Carlos G. Grijalva, Todd W. Rice, Ian D. Jones, William B. Stubblefield, Adrienne Baughman, Kelsey N. Womack, Christopher J. Lindsell, Kimberly W. Hart, Yuwei Zhu, Meagan Stephenson, Stephanie J. Schrag, Miwako Kobayashi, Jennifer R. Verani, Manish M. Patel, IVY Network Investigators, IVY Network, Nicole Calhoun, Kempapura Murthy, Judy Herrick, Amanda McKillop, Eric Hoffman, Martha Zayed, Michael Smith, Natalie Settele, Jason Ettlinger, Elisa Priest, Jennifer Thomas, Alejandro Arroliga, Madhava Beeram, Ryan Kindle, Lori-Ann Kozikowski, Lesley De Souza, Scott Ouellette, Sherell Thornton-Thompson, Patrick Tyler, Omar Mehkri, Kiran Ashok, Susan Gole, Alexander King, Bryan Poynter, Nicholas Stanley, Audrey Hendrickson, Ellen Maruggi, Tyler Scharber, Jeffrey Jorgensen, Robert Bowers, Jennifer King, Valerie Aston, Brent Armbruster, Richard E. Rothman, Rahul Nair, Jen-Ting (Tina) Chen, Sarah Karow, Emily Robart, Paulo Nunes Maldonado, Maryiam Khan, Preston So, Joe Levitt, Cynthia Perez, Anita Visweswaran, Jonasel Roque, Adreanne Rivera, Trevor Frankel, Michelle Howell, Jennifer Friedel, Jennifer Goff, David Huynh, Michael Tozier, Conner Driver, Michael Carricato, Alexandra Foster, Paul Nassar, Lori Stout, Zita Sibenaller, Alicia Walter, Jasmine Mares, Logan Olson, Bradley Clinansmith, Carolina Rivas, Hayley Gershengorn, EJ McSpadden, Rachel Truscon, Anne Kaniclides, Lara Thomas, Ramsay Bielak, Weronika Damek Valvano, Rebecca Fong, William J. Fitzsimmons, Christopher Blair, Andrew L. Valesano, Julie Gilbert, Christine D. Crider, Kyle A. Steinbock, Thomas C. Paulson, Layla A. Anderson, Christy Kampe, Jakea Johnson, Rendie McHenry, Marcia Blair, Douglas Conway, Mary LaRose, Leigha Landreth, Madeline Hicks, Lisa Parks, Jahnavi Bongu, David McDonald, Candice Cass, Sondra Seiler, David Park, Tiffany Hink, Meghan Wallace, Carey-Ann Burnham, Olivia G. Arter

**Affiliations:** ^1^CDC COVID-19 Response Team; ^2^Vanderbilt University Medical Center, Nashville, Tennessee; ^3^University of Colorado School of Medicine, Aurora, Colorado; ^4^University of Iowa, Iowa City, Iowa; ^5^Baylor Scott & White Health, Temple, Texas; ^6^Texas A&M University College of Medicine, Temple, Texas; ^7^Beth Israel Deaconess Medical Center, Boston, Massachusetts; ^8^Wake Forest University Baptist Medical Center, Winston-Salem, North Carolina; ^9^Johns Hopkins Hospital, Baltimore, Maryland; ^10^Hennepin County Medical Center, Minneapolis, Minnesota; ^11^Montefiore Healthcare Center, Albert Einstein College of Medicine, Bronx, New York; ^12^University of Washington School of Medicine, Seattle, Washington; ^13^Baystate Medical Center, Springfield, Massachusetts; ^14^Intermountain Medical Center and University of Utah, Salt Lake City, Utah; ^15^University of Michigan School of Public Health, Ann Arbor, Michigan; ^16^Oregon Health & Science University Hospital, Portland, Oregon; ^17^Emory University School of Medicine, Atlanta, Georgia; ^18^Cleveland Clinic, Cleveland, Ohio; ^19^Stanford University School of Medicine, Palo Alto, California; ^20^Ronald Reagan-UCLA Medical Center, Los Angeles, California; ^21^University of Miami, Miami, Florida; ^22^Washington University, St. Louis, Missouri; ^23^Ohio State University Wexner Medical Center, Columbus, Ohio; ^24^University of Michigan School of Medicine, Ann Arbor, Michigan.; Baylor Scott & White Health; Baylor Scott & White Health; Baylor Scott & White Health; Baylor Scott & White Health; Baylor Scott & White Health; Baylor Scott & White Health; Baylor Scott & White Health; Baylor Scott & White Health; Baylor Scott & White Health; Baylor Scott & White Health; Baylor Scott & White Health; Baylor Scott & White Health; Baylor Scott & White Health; Baystate Medical Center; Baystate Medical Center; Baystate Medical Center; Baystate Medical Center; Baystate Medical Center; Beth Israel Deaconess Medical Center; Cleveland Clinic; Cleveland Clinic; Cleveland Clinic; Cleveland Clinic; Cleveland Clinic; Emory University; Hennepin County Medical Center; Hennepin County Medical Center; Hennepin County Medical Center; Intermountain Medical Center; Intermountain Medical Center; Intermountain Medical Center; Intermountain Medical Center; Intermountain Medical Center; Johns Hopkins University; Montefiore Medical Center; Montefiore Medical Center; Ohio State University; Ohio State University; Ohio State University; Ohio State University; Ohio State University; Stanford University; Stanford University; Stanford University; Stanford University; University of California, Los Angeles; University of California, Los Angeles; UCHealth University of Colorado Hospital; UCHealth University of Colorado Hospital; UCHealth University of Colorado Hospital; UCHealth University of Colorado Hospital; UCHealth University of Colorado Hospital; UCHealth University of Colorado Hospital; UCHealth University of Colorado Hospital; UCHealth University of Colorado Hospital; University of Iowa; University of Iowa; University of Iowa; University of Iowa; University of Iowa; University of Iowa; University of Iowa; University of Miami; University of Miami; University of Michigan; University of Michigan; University of Michigan; University of Michigan; University of Michigan; University of Michigan; University of Michigan; University of Michigan; University of Michigan; University of Michigan; University of Michigan; University of Washington; University of Washington; University of Washington; University of Washington; Vanderbilt University Medical Center; Vanderbilt University Medical Center; Vanderbilt University Medical Center; Vanderbilt University Medical Center; Vanderbilt University Medical Center; Wake Forest University; Wake Forest University; Wake Forest University; Wake Forest University; Washington University; Washington University; Washington University; Washington University; Washington University; Washington University; Washington University; Washington University; Washington University

Real-world evaluations have demonstrated high effectiveness of vaccines against COVID-19–associated hospitalizations (*1*–*4*) measured shortly after vaccination; longer follow-up is needed to assess durability of protection. In an evaluation at 21 hospitals in 18 states, the duration of mRNA vaccine (Pfizer-BioNTech or Moderna) effectiveness (VE) against COVID-19–associated hospitalizations was assessed among adults aged ≥18 years. Among 3,089 hospitalized adults (including 1,194 COVID-19 case-patients and 1,895 non–COVID-19 control-patients), the median age was 59 years, 48.7% were female, and 21.1% had an immunocompromising condition. Overall, 141 (11.8%) case-patients and 988 (52.1%) controls were fully vaccinated (defined as receipt of the second dose of Pfizer-BioNTech or Moderna mRNA COVID-19 vaccines ≥14 days before illness onset), with a median interval of 65 days (range = 14–166 days) after receipt of second dose. VE against COVID-19–associated hospitalization during the full surveillance period was 86% (95% confidence interval [CI] = 82%–88%) overall and 90% (95% CI = 87%–92%) among adults without immunocompromising conditions. VE against COVID-19– associated hospitalization was 86% (95% CI = 82%–90%) 2–12 weeks and 84% (95% CI = 77%–90%) 13–24 weeks from receipt of the second vaccine dose, with no significant change between these periods (p = 0.854). Whole genome sequencing of 454 case-patient specimens found that 242 (53.3%) belonged to the B.1.1.7 (Alpha) lineage and 74 (16.3%) to the B.1.617.2 (Delta) lineage. Effectiveness of mRNA vaccines against COVID-19–associated hospitalization was sustained over a 24-week period, including among groups at higher risk for severe COVID-19; ongoing monitoring is needed as new SARS-CoV-2 variants emerge. To reduce their risk for hospitalization, all eligible persons should be offered COVID-19 vaccination.

Evaluations of authorized mRNA COVID-19 vaccines (Pfizer-BioNTech and Moderna) have consistently demonstrated high VE across diverse populations (*1*,*5*). Because COVID-19 vaccines were initially authorized in the United States in December 2020, evaluations of real-world effectiveness have been subject to a short period of postvaccination follow-up. Monitoring durability of protection after COVID-19 vaccination can help determine whether booster vaccines might be indicated, particularly with continued emergence of new variants that might overcome vaccine-induced immunity. In real-world settings, durability of protection has commonly been measured by comparing the odds of vaccination in laboratory-confirmed case-patients and control-patients who tested negative for infection, by time since vaccination (*6*,*7*).

During March 11–July 14, 2021, adults aged ≥18 years admitted to 21 hospitals in 18 states were included in an analysis of durability of vaccine-induced protection. Case-patients had COVID-19–like illness^†^ and had received a positive SARS-CoV-2 reverse transcription–polymerase chain reaction (RT-PCR) or antigen test result. A first group of hospital-based control-patients had COVID-19–like illness and had negative SARS-COV-2 results by all tests, including at least one RT-PCR test. A second hospital-based control group of patients without COVID-19–like illness (and therefore unlikely to be hospitalized for COVID-19–like illness) was also enrolled (*4*). This second control group also received negative SARS-CoV-2 results by all tests, including at least one RT-PCR test. Eligibility for enrollment as a case-patient or one of these controls required SARS-CoV-2 testing within 10 days of symptom onset and hospital admission within 14 days of symptom onset. Final case/control status was determined using clinical testing results and central laboratory RT-PCR testing of upper respiratory specimens (nasal swabs or saliva) performed at a central laboratory (Vanderbilt University Medical Center, Nashville, Tennessee) (*4*). Specimens positive for SARS-CoV-2 with cycle threshold values <32 were sent to University of Michigan (Ann Arbor, Michigan) for whole genome sequencing and SARS-CoV-2 lineage determination (*4*).

Patients or their proxies were interviewed about baseline demographic characteristics, clinical history (including COVID-19–like signs or symptoms experienced and date of illness onset), and history of COVID-19 vaccination. Vaccine was considered to have been administered if vaccination dates and product names were verified through medical records, state immunization registries, vaccination record cards, or provider or pharmacy records, or if plausibly reported by patient or proxy with date and location of vaccination. A patient was considered to be fully vaccinated if both doses of an authorized mRNA COVID-19 vaccine were administered, with the second dose received ≥14 days before illness onset.^§^ Participants were excluded from this analysis if they received only 1 dose of an mRNA COVID-19 vaccine, received 2 doses with the second dose <14 days before illness onset, received a non-mRNA COVID-19 vaccine, or received mixed products of an mRNA COVID-19 vaccine (i.e., a different product for each dose).

Vaccine effectiveness against COVID-19–associated hospitalization was estimated using logistic regression, comparing the odds of being fully vaccinated versus unvaccinated between case-patients and controls (including both control groups) using the equation VE = 100 × (1 – odds ratio) (*1*). VE over the full surveillance period was assessed, as well as among those with illness onset during March–May and June–July 2021, because of increased circulation of Delta variants in the United States during the latter period (*8*). Models were adjusted for potential confounders, including admission date (biweekly intervals), U.S. Department of Health and Human Services region, age, sex, and race/ethnicity. Time-varying VE models were then constructed. First, a binary model was constructed by adding a categorical term (2–12 weeks versus 13–24 weeks) for interval from receipt of the second vaccine dose (among vaccinated participants) and illness onset. Unvaccinated patients were assigned values of zero days since vaccination. In additional analyses, other specifications of time were considered, including using linear and natural cubic spline terms. Bootstrapping with 1,000 replications was used to estimate 95% CIs. Subgroup analyses included adults aged ≥65 years, patients with immunocompromising conditions,^¶^ and patients with three or more categories of chronic medical conditions. A sensitivity analysis was also performed including each of the two control groups in models rather than combining them. Significance of association between VE and time since vaccination was assessed using a likelihood-ratio chi-square test with p-values <0.05 considered statistically significant. Analyses were conducted using R statistical software (version 4.0.3; R Foundation). This activity was determined to be public health surveillance by each participating site and CDC and was conducted consistent with applicable federal law and CDC policy.**

After excluding 722 ineligible patients (461 who were not fully vaccinated or unvaccinated, 127 who received a non-mRNA COVID-19 vaccine or mixed vaccines, and 134 who did not meet other inclusion criteria), 3,089 patients were included in the final analysis (1,194 case-patients and 1,895 in the combined control groups) (Table). The median patient age was 59 years (interquartile range = 46–69 years), 48.7% were female, 56.7% were non-Hispanic White, and 21.1% had an immunocompromising condition. Among case-patients, 141 (11.8%) were fully vaccinated as were 988 (52.1%) controls. Among 454 case-patient specimens with SARS-CoV-2 lineage determined, 242 (53.3%) were identified as Alpha and 74 (16.3%) as Delta (Figure 1). Delta variants became the dominant virus in mid-June. Overall VE against hospitalization for COVID-19 was 86% (95% CI = 82%–88%) over the full surveillance period, including 90% (95% CI = 87%–92%) among patients without immunocompromising conditions and 63% (95% CI = 44%–76%) among patients with immunocompromising conditions. VE among patients with illness onset during March–May was 87% (95% CI = 83%–90%), and among those with illness onset during June–July was 84% (95% CI = 79%–89%). In models considering time since vaccination, VE was 86% (95% CI = 82%–90%) during the 2–12 weeks after the second vaccine dose and 84% (95% CI = 77%–90%) 13–24 weeks after the second dose; there was no significant difference in VE between these two periods (p = 0.854). Models treating time since vaccination as linear and as a natural cubic spline with a knot at the median and boundary knots at the 10th and 90th percentiles also showed no significant change in VE over a 24-week period (linear p = 0.400, spline p = 0.234) (Supplementary Figure, https://stacks.cdc.gov/view/cdc/108758). No significant change in VE over a 24-week period was observed within subgroups (all p>0.05) (Figure 2). In sensitivity analyses, results were similar using individual control groups and combined controls.

**TABLE:** Characteristics of COVID-19 case-patients and controls among hospitalized adults — 21 academic medical centers in 18 states, March–July 2021

Characteristic	No. (%)			P-value*
	Overall (N = 3,089)	Cases (n = 1,194)	Controls (n = 1,895)	
**Median age, yrs (IQR)**	59 (46–69)	56 (42–66)	62 (48–71)	<0.001
**Age group, yrs**				<0.001
18–49	950 (30.8)	445 (37.3)	505 (26.7)	
50–64	1,008 (32.6)	424 (35.5)	584 (30.8)	
≥65	1,131 (36.6)	325 (27.2)	806 (42.5)	
**Sex**				
Female	1,504 (48.7)	580 (48.6)	924 (48.8)	0.921
**Race/Ethnicity**				<0.001
White, non-Hispanic	1,752 (56.8)	548 (45.9)	1,204 (63.5)	
Black, non-Hispanic	693 (22.4)	312 (26.1)	381 (20.1)	
Hispanic, any race	467 (15.1)	245 (20.5)	222 (11.7)	
Other, non-Hispanic	140 (4.5)	67 (5.6)	73 (3.9)	
Unknown	37 (1.2)	22 (1.8)	15 (0.8)	
**Region^†^**				<0.001
Northeast	432 (14.0)	165 (13.8)	267 (14.1)	
South	1,151 (37.3)	459 (38.4)	692 (36.5)	
Midwest	818 (26.5)	265 (22.2)	553 (29.2)	
West	688 (22.3)	305 (25.5)	383 (20.2)	
**Resident in long-term care facility (100 unknown)**	141 (4.7)	29 (2.5)	112 (6.1)	<0.001
**Previous hospitalization in last year (231 unknown)**	1,297 (45.4)	319 (30.0)	978 (54.5)	<0.001
**No. of baseline conditions (2 unknown)^§^**				<0.001
0	552 (17.9)	301 (25.2)	251 (13.3)	
1	736 (23.8)	310 (26.0)	426 (22.5)	
2	766 (24.8)	260 (21.8)	506 (26.7)	
≥3	1,033 (33.5)	322 (27.0)	711 (37.5)	
**Specific chronic conditions**				
Cardiovascular disease (1 unknown)	1,900 (61.5)	647 (54.2)	1,253 (66.2)	<0.001
Pulmonary disease (1 unknown)	804 (26.0)	257 (21.5)	547 (28.9)	<0.001
Diabetes mellitus (1 unknown)	952 (30.8)	348 (29.2)	604 (31.9)	0.108
Immunocompromising condition*(2 unknown)^¶^	652 (21.1)	205 (17.2)	447 (23.6)	<0.001
Fully vaccinated**	1,129 (36.6)	141 (11.8)	988 (52.1)	<0.001
**Vaccine product received (among fully vaccinated persons)**				0.030
Pfizer-BioNTech	666 (59.0)	95 (67.4)	571 (57.8)	
Moderna	463 (41.0)	46 (32.6)	417 (42.2)	
If fully vaccinated, median (IQR) days from second vaccine dose and onset of symptoms	65 (41–93)	60 (36–94)	66 (42–93)	0.509

**Abbreviation:** IQR = interquartile range.

* P-values determined using the Wilcoxon rank-sum test for continuous variables and by chi-square test of independence for categorical variables.

^†^ Hospitals by region included *Northeast*: Baystate Medical Center (Springfield, Massachusetts), Beth Israel Deaconess Medical Center (Boston, Massachusetts), Montefiore Medical Center (Bronx, New York); *South*: Vanderbilt University Medical Center (Nashville, Tennessee), University of Miami Medical Center (Miami, Florida), Emory University Medical Center (Atlanta, Georgia), Johns Hopkins Hospital (Baltimore, Maryland), Wake Forest University Baptist Medical Center (Winston-Salem, North Carolina), Baylor Scott and White Health (Temple, Texas); *Midwest*: University of Iowa Hospitals (Iowa City, Iowa), University of Michigan Hospital (Ann Arbor, Michigan), Hennepin County Medical Center (Minneapolis, Minnesota), Barnes-Jewish Hospital (St. Louis, Missouri), Cleveland Clinic (Cleveland, Ohio), Ohio State University Wexner Medical Center (Columbus, Ohio); *West*: Stanford University Medical Center (Stanford, California), UCLA Medical Center (Los Angeles, California), UCHealth University of Colorado Hospital (Aurora, Colorado), Oregon Health & Science University Hospital (Portland, Oregon), Intermountain Medical Center (Murray, Utah), University of Washington (Seattle, Washington).

**^§^** Chronic condition categories included the following: cardiovascular disease, neurologic disease, pulmonary disease, gastrointestinal disease, endocrine disease, renal disease, hematologic disease, malignancy, immunosuppression not captured in other categories, autoimmune condition, or other condition (sarcoidosis, amyloidosis, or unintentional weight loss ≥10 pounds in the last 90 days).

^¶^ Immunocompromising conditions included having one or more of the following: active solid organ cancer (active cancer defined as treatment for the cancer or newly diagnosed cancer in the past 6 months), active hematologic cancer (such as leukemia, lymphoma, or myeloma), HIV infection without AIDS, AIDS, congenital immunodeficiency syndrome, previous splenectomy, previous solid organ transplant, immunosuppressive medication, systemic lupus erythematosus, rheumatoid arthritis, psoriasis, scleroderma, or inflammatory bowel disease, including Crohn’s disease or ulcerative colitis.

** COVID-19 vaccination status included unvaccinated, defined as no receipt of any SARS CoV-2 vaccine, and fully vaccinated, defined as receipt of both doses of a 2-dose mRNA vaccine with the second dose received ≥14 days before illness onset.

**FIGURE 1:**
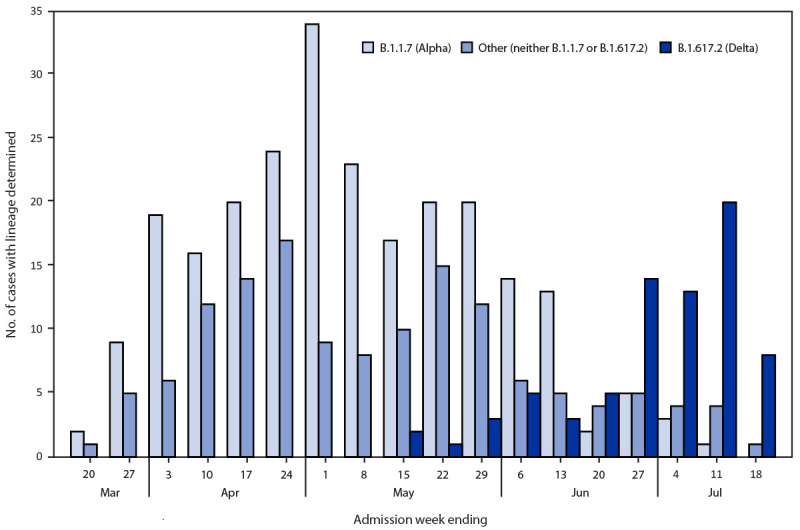
Whole genome sequencing lineage determination among adults hospitalized with COVID-19 — 21 academic medical centers in 18 states,*^,†^ March–July 2021 * Specimens with SARS-CoV-2 detected by reverse transcription–polymerase chain reaction and with a cycle threshold <32 for at least one of two nucleocapsid gene targets tested underwent whole genome sequencing. SARS-CoV-2 lineages were assigned with >80% coverage using Pangolin genomes. Results are presented for B.1.1.7 (Alpha) variants, B.1.617.2 (Delta) variants, and other (neither B.1.1.7 or B.1.617.2) variant with lineage determined by whole genome sequencing. Of 74 Delta variants sequenced, four belonged to the AY.3 Delta sublineage. The histogram provides the number of viruses sequenced by week of hospital admission. ^†^ Hospitals by region included *Northeast*: Baystate Medical Center (Springfield, Massachusetts), Beth Israel Deaconess Medical Center (Boston, Massachusetts), Montefiore Medical Center (Bronx, New York); *South*: Vanderbilt University Medical Center (Nashville, Tennessee), University of Miami Medical Center (Miami, Florida), Emory University Medical Center (Atlanta, Georgia), Johns Hopkins Hospital (Baltimore, Maryland), Wake Forest University Baptist Medical Center (Winston-Salem, North Carolina), Baylor Scott and White Health (Temple, Texas); *Midwest*: University of Iowa Hospitals (Iowa City, Iowa), University of Michigan Hospital (Ann Arbor, Michigan), Hennepin County Medical Center (Minneapolis, Minnesota), Barnes-Jewish Hospital (St. Louis, Missouri), Cleveland Clinic (Cleveland, Ohio), Ohio State University Wexner Medical Center (Columbus, Ohio); *West*: Stanford University Medical Center (Stanford, California), UCLA Medical Center (Los Angeles, California), UCHealth University of Colorado Hospital (Aurora, Colorado), Oregon Health & Science University Hospital (Portland, Oregon), Intermountain Medical Center (Murray, Utah), University of Washington (Seattle, Washington).

**FIGURE 2:**
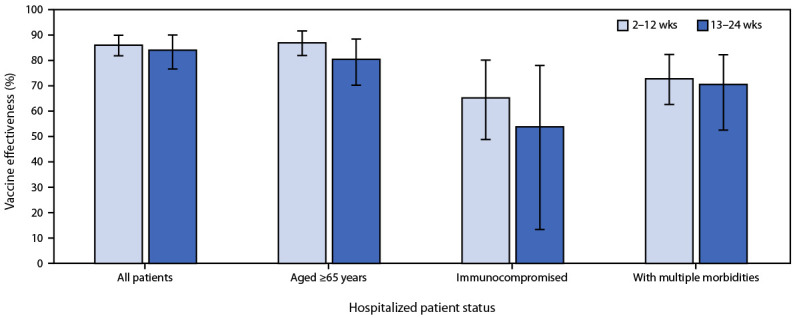
Sustained vaccine effectiveness* against COVID-19 among hospitalized adults, by patient status^†^^,^^§^ and interval since vaccination — 21 medical centers in 18 states,^¶^ March–July 2021 **Abbreviation:** VE = vaccine effectiveness. * VE was estimated using logistic regression comparing the odds of being fully vaccinated with an authorized mRNA COVID-19 vaccine with being unvaccinated in case patients and controls using the equation VE = 100 × (1 – odds ratio). Models were adjusted for date of hospital admission (biweekly intervals), U.S. Department of Health and Human Services region of hospital, age group (18–49, 50–64, or ≥65 years), sex, and race/ethnicity (non-Hispanic White, non-Hispanic Black, Hispanic of any race, non-Hispanic Other, or unknown). Analyses restricted to adults aged ≥65 years adjusted for age in years as a continuous variable. Binary time since second dose of mRNA vaccine was added to the model with results for 2–12 weeks and 13–24 weeks shown. 95% confidence intervals shown by error bars. ^†^ Immunocompromising conditions included having one or more of the following: active solid organ cancer (active cancer defined as treatment for the cancer or newly diagnosed cancer in the past 6 months), active hematologic cancer (such as leukemia, lymphoma, or myeloma), HIV infection without AIDS, AIDS, congenital immunodeficiency syndrome, previous splenectomy, previous solid organ transplant, immunosuppressive medication, systemic lupus erythematosus, rheumatoid arthritis, psoriasis, scleroderma, or inflammatory bowel disease, including Crohn’s disease or ulcerative colitis. **^§^** Multiple morbidities were defined as having chronic conditions within three or more of the following condition categories: cardiovascular disease, neurologic disease, pulmonary disease, gastrointestinal disease, endocrine disease, renal disease, hematologic disease, malignancy, immunosuppression not captured in other categories, autoimmune condition, or other condition (sarcoidosis, amyloidosis, or unintentional weight loss ≥10 pounds in the last 90 days). ^¶^ Hospitals by region included *Northeast*: Baystate Medical Center (Springfield, Massachusetts), Beth Israel Deaconess Medical Center (Boston, Massachusetts), Montefiore Medical Center (Bronx, New York); *South*: Vanderbilt University Medical Center (Nashville, Tennessee), University of Miami Medical Center (Miami, Florida), Emory University Medical Center (Atlanta, Georgia), Johns Hopkins Hospital (Baltimore, Maryland), Wake Forest University Baptist Medical Center (Winston-Salem, North Carolina), Baylor Scott and White Health (Temple, Texas); *Midwest*: University of Iowa Hospitals (Iowa City, Iowa), University of Michigan Hospital (Ann Arbor, Michigan), Hennepin County Medical Center (Minneapolis, Minnesota), Barnes-Jewish Hospital (St. Louis, Missouri), Cleveland Clinic (Cleveland, Ohio), Ohio State University Wexner Medical Center (Columbus, Ohio); *West*: Stanford University Medical Center (Stanford, California), UCLA Medical Center (Los Angeles, California), UCHealth University of Colorado Hospital (Aurora, Colorado), Oregon Health & Science University Hospital (Portland, Oregon), Intermountain Medical Center (Murray, Utah), University of Washington (Seattle, Washington).

## Discussion

In a multistate network that enrolled adults hospitalized during March–July 2021, effectiveness of 2 doses of mRNA vaccine against COVID-19–associated hospitalization was sustained over a follow-up period of 24 weeks (approximately 6 months). These findings of sustained VE were consistent among subgroups at highest risk for severe outcomes from COVID-19, including older adults, adults with three or more chronic medical conditions, and those with immunocompromising conditions. Overall VE in adults with immunocompromising conditions was lower than that in those without immunocompromising conditions but was sustained over time in both populations.

These data provide evidence for sustained high protection from severe COVID-19 requiring hospitalization for up to 24 weeks among fully vaccinated adults, which is consistent with data demonstrating mRNA COVID-19 vaccines have the capacity to induce durable immunity, particularly in limiting the severity of disease (*9**,**10*). Alpha variants were the predominant viruses sequenced, although Delta variants became dominant starting in mid-June, consistent with national surveillance data (*8*). Because of limited sequenced virus, Delta-specific VE was not assessed. VE was similar during June–July when circulation of Delta increased in the United States compared with VE during March–May when Alpha variants predominated, although further surveillance is needed.

The findings in this report are subject to at least six limitations. First, the follow-up period was limited to approximately 24 weeks since receipt of full vaccination because of the recent authorization of mRNA COVID-19 vaccines in the United States. Additional analyses with longer duration of follow-up since vaccination are warranted. Second, effectiveness over time from authorized non-mRNA COVID-19 vaccines, including Janssen’s (Johnson & Johnson) vaccine product, was not assessed because of limited use of this vaccine during the surveillance period. Third, time-varying VE was not assessed by lineage because of sample size. Fourth, residual confounding might have been present, although the analysis adjusted for potential confounders, including calendar time and patient age. Fifth, this analysis did not consider VE over time among persons aged <18 years or partially vaccinated persons. Finally, the current analysis only included hospitalized adults and did not include persons with asymptomatic SARS-CoV-2 infection or COVID-19 who did not require hospitalization.

Protection against severe COVID-19 resulting in hospitalization was sustained through 24 weeks after vaccination with mRNA COVID-19 vaccines. To reduce their risk for hospitalization, all eligible persons should be offered COVID-19 vaccination. Continued monitoring of VE against infection and severe disease is needed as the elapsed time since vaccination increases and new SARS-CoV-2 variants emerge.

**Summary**

**What is already known about this topic?**

COVID-19 mRNA vaccines provide strong protection against severe COVID-19; however, the duration of protection is uncertain.

**What is added by this report?**

Among 1,129 patients who received 2 doses of a mRNA vaccine, no decline in vaccine effectiveness against COVID-19 hospitalization was observed over 24 weeks. Vaccine effectiveness was 86% 2–12 weeks after vaccination and 84% at 13–24 weeks. Vaccine effectiveness was sustained among groups at risk for severe COVID-19.

**What are the implications for public health practice?**

mRNA vaccine effectiveness against COVID-19–associated hospitalizations was sustained over 24 weeks; ongoing monitoring is needed as new SARS-CoV-2 variants emerge. To reduce hospitalization, all eligible persons should be offered COVID-19 vaccination.

## Footnotes

* These authors contributed equally to this report.

† COVID-19–like illness was defined as having one or more of the following: fever, cough, shortness of breath, loss of taste, loss of smell, use of respiratory support for the acute illness, or new pulmonary findings on chest imaging consistent with pneumonia.

§ The date of illness onset was used for cases and controls with COVID-19–like illness with median value imputed if missing. For controls without COVID-19–like illness, the date of admission minus the median number of days between illness onset and admission for patients with COVID-19 was used for a date of illness onset, also referred to as “illness onset” for this report.

¶ Immunocompromising conditions included having one or more of the following: active solid organ cancer (active cancer defined as treatment for the cancer or newly diagnosed cancer in the past 6 months), active hematologic cancer (such as leukemia, lymphoma, or myeloma), HIV infection without AIDS, AIDS, congenital immunodeficiency syndrome, previous splenectomy, previous solid organ transplant, immunosuppressive medication, systemic lupus erythematosus, rheumatoid arthritis, psoriasis, scleroderma, or inflammatory bowel disease, including Crohn’s disease or ulcerative colitis.

** 45 C.F.R. part 46.102(l)(2), 21 C.F.R. part 56; 42 U.S.C. Sect 241(d); 5 U.S.C. Sect 552a; 44 U.S.C. Sect 3501 et seq.
